# One-Pot Synthesis of Alkyl Functionalized Reduced Graphene Oxide Nanocomposites as the Lubrication Additive Enabling Enhanced Tribological Performance

**DOI:** 10.3390/molecules29092004

**Published:** 2024-04-26

**Authors:** Guangfa Zhang, Chao Zhu, Yehai Yan, Jian Cui, Jingxian Jiang

**Affiliations:** 1 Key Laboratory of Rubber-Plastics, Ministry of Education/Shandong Provincial Key Laboratory of Rubber-Plastics, School of Polymer Science and Engineering, Qingdao University of Science and Technology, Qingdao 266042, China; 2 School of Chemical and Environmental Engineering, Shanghai Institute of Technology, Shanghai 201418, China

**Keywords:** bromooctadecane, lubricant additives, one-step approach, friction reducing, wear resistance

## Abstract

Recently, aiming for the enhanced dispersibility of graphene-based nanomaterials in lubricating oil matrices to serve as highly efficient lubricant additives, numerous modification approaches have been extensively studied. However, these previous modification routes usually involve a tedious multistep modification process or multitudinous toxic reagents, restricting their extensive practical application. In this work, novel graphene oxide (GO) nanoadditives (RGO-*g*-BO) featuring excellent durable dispersion capability and remarkable tribological performance were successfully prepared via an environmentally friendly one-step approach consisting of surface grafting of long-chain bromooctadecane (BO) and in situ chemical reduction. Benefiting from the greatly improved lipophilicity (resulting from the introduction of hydrophobic long-chain alkane groups and chemical reduction), along with the miniaturization effect, RGO-*g*-BO exhibits superior long-term dispersion stability in the finished oil. Moreover, the tribological properties results demonstrated that the finished oil filled with RGO-*g*-BO nanolubricants achieved an outstanding friction-reducing and antiwear performance. Particularly, under the optimum content of RGO-*g*-BO (as low as 0.005 wt%), the friction coefficient as well as the wear volume of the composite finished oil were greatly reduced by 13% and 53%, respectively, as compared with nascent finished oil. Therefore, in view of the advantages of low-cost, one-step facile synthesis, desirable dispersion capability, and remarkable tribological performance, RGO-*g*-BO holds great prospects as a highly efficient lubrication additive in the tribology field.

## 1. Introduction

Nowadays, with the rapid development of industry and the extensive use of various machinery, the friction and wear occurred between adjacent mechanical surfaces have received considerable attention [1,2,3]. This undesired process will inevitably cause massive energy dissipation and reduced service life of mechanic parts, thus greatly increasing the running cost and even leading to major production accidents [4,5,6]. To address this issue, different kinds of lubricating oils have been exploited and applied in recent years. However, environmental protection and energy saving placed higher application requirements for lubricants in friction-reducing and wear-resistance performance, especially for modern mechanical equipment. Hence, pure base oil does not easily meet the requirements of the modern industry, so solid nanoadditives such as ZnO, CuO, Fe_3_O_4_, SiO_2_, and CeO_2_ have been widely explored in last decade, which are utilized as additives and added to lubricating oil to enhance its lubrication performance [7]. These traditional nanoadditives suffer from significant defects, including a complex preparation process and inferior dispersion capacity in lube oils, as well as poor lubricating stability, which severely restrict their further development [6]. Therefore, high-performance lubricant additives with more facile synthesis technology and excellent compatibility in the lube oil matrix are highly required.

Graphene featuring an ultrathin 2D layered structure, a new star of the carbon material family, has shown various applications, such as composites, sensors, batteries, biological instruments, etc. [8,9,10,11,12,13]. Owing to its unmatched advantages such as distinct single-layer structure, remarkable mechanical strength, excellent chemical and thermal stability, and low resistance to shear (self-lubricating property), graphene is regarded as a promising candidate for lubrication material [14,15,16]. The physical lubrication mechanisms of graphene can be described as follows: Due to the smaller size and ultrathin lamellar structure, graphene nanoplatelets can easily enter the rubbing interfaces between the neighboring friction pairs and form a robust graphene-based tribofilm, inhibiting the direct asperity contact of rubbing surfaces and hence dramatically decreasing the friction and wear during the friction process [14,17,18]. Numerous approaches have been adopted to develop high-efficiency graphene-based lubricant nanoadditives for improving friction-reducing and antiwear performance [19,20,21]. Despite these advances, the aggregation behavior of nascent graphene flakes in the lubricating oil matrix hinders its wide application in the tribology field [22]. To address this thorny problem, researchers have proposed two main strategies to elevate the dispersion capacity of original graphene, namely the introduction of molecular dispersants and surface chemical modification [23,24,25]. Nevertheless, these approaches display some obvious shortcomings; for example, (I) due to the higher shear stresses and higher friction temperatures at the rubbing surfaces, the incorporated molecular additives easily degrade during the friction process; (II) it is very difficult to perform covalently functional modification (e.g., surface grafting) owing to the inherent high chemical inertness of pure graphene (without any active functional groups) [26,27].

Recently, graphene oxide (GO) has been increasingly regarded as a promising and effective lubricant additive due to its fascinating distinct advantages [28,29,30]: (a) The numerous oxygenated functional groups (including hydroxyl, carboxyl, and epoxy groups) on the GO base and edge ensure a key chemical basis and facilitate further surface chemical modification [31]; (b) it possesses an intriguing 2D ultrathin laminated structure and outstanding mechanical robustness; (c) more importantly, it has great practical application prospects because of both the mature manufacturing technology and cost-effective features of GO. Nevertheless, given the abundant of oxygen-containing groups on the GO surface, which can not only significantly reduce its intrinsic thermal stability but also greatly decrease its dispersibility in the non-polar lubricating oil matrix, pure GO cannot be utilized directly as an additive in lubricant oils [32,33]. To overcome this limitation, considerable efforts have been devoted in recent years to improve its lipophilicity and enhance its dispersion stability and durability in lubricant oils [34,35,36]. Among them, the surface grafting of long-chain alkylated functional groups onto GO (yielding alkylated GO) and the removal of oxygen groups by thermal or chemical reduction are considered the two most efficient strategies to fulfill this purpose [30,32,37,38]. However, the previously reported methods usually involve tedious and complex preparation processes or the utilization of massive toxic chemicals.

**Scheme 1:**
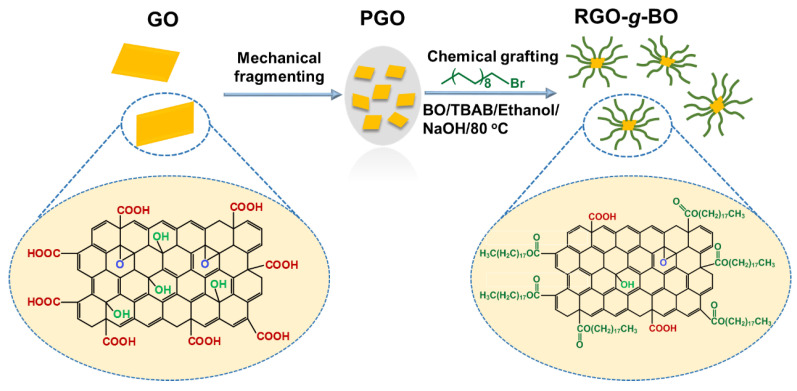
Schematic illustration of the preparation process of RGO-*g*-BO nanosheets.

Herein, we successfully prepared a novel long-chain alkyl functionalized reduced graphene oxide (RGO-*g*-BO) nanomaterial through a facile and green one-pot synthesis technology. As presented in Scheme 1, the preparation process mainly contains two key steps, namely the surface grafting of 1-bromooctadecane (BO) and controllable, partial chemical reduction with sodium hydroxide. The structural characteristics, chemical composition, micromorphology, and particle size distribution of the resultant RGO-*g*-BO were systematically examined. As expected, the as-obtained RGO-*g*-BO displayed excellent thermal and dispersion stability in the finished oil. The tribological performance of finished oils filled with different mass fractions of RGO-*g*-BO was comprehensively assessed with a reciprocating friction and wear method. Combining the exploration of the morphology and components of the wear scar after friction, the underlying friction-reducing and anti-wear mechanisms were investigated as well.

## 2. Results and Discussion

### 2.1. Chemical Composition and Structural Characterization

**Figure 1:**
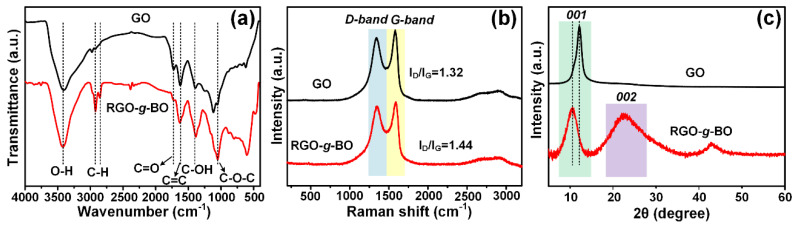
(**a**) FTIR spectra, (**b**) Raman spectra, and (**c**) XRD patterns of GO and RGO−*g*−BO.

In this work, the long-chain alkyl functionalized reduced graphene oxide nanocomposites RGO-*g*-BO were fabricated via a facile one-pot method (Scheme 1). To explore the chemical composition and structural evolution of GO and RGO-*g*-BO during the fabrication process, FTIR, Raman spectra, and XRD analysis of these materials were comprehensively measured. Figure 1a shows the FTIR results of GO and RGO-*g*-BO. For GO, the peaks located at ~3400 and 1404 cm^−1^ are attributed to the stretching vibration of hydroxyl (O-H) and the in-plane bending vibration of carboxyl C-OH, respectively. Moreover, the characteristic peaks of 1720, 1650, and 1050 cm^−1^ can be ascribed to the stretching vibrations of C=O, C=C, and C-O-C, respectively. These results distinctly suggest that abundant oxygenated functional groups (such as hydroxyl, carboxyl, and epoxy groups) were successfully decorated/immobilized onto the GO sheets, including the basal plane and edge locations (as shown in Scheme 1) [39]. In contrast, as for RGO-*g*-BO, the emerging peaks at 2922 and 2854 cm^−1^ are clearly observed, which are associated with the stretching vibrations of the long-chain alkyl groups (i.e., -CH_2_ and -CH_3_). Consequently, it is a reasonable conclusion that BO components were successfully grafted onto the surface of GO through the facile grafting reaction. Moreover, as compared to GO, both the peak strength of carboxyl groups (C=O, 1720 cm^−1^) and the peak breadth of the hydroxyl group (3400 cm^−1^) in RGO-*g*-BO display an obvious decrease. The above phenomena reveal that the majority of oxygen-containing functional groups in GO sheets were effectively removed owing to the chemical reduction process (with NaOH) and in situ thermal reduction (during the grafting reaction step).

In addition, Raman spectroscopy is an effective tool that is commonly used to determine the structural integrity of carbon-based materials (such as graphene, graphene oxide, and carbon nanotubes) [40,41]. Figure 1b shows the Raman spectra of GO and RGO-*g*-BO. Notably, there are two typical peaks for the tested samples: the D band (at ~1350 cm^−1^) and the G band (at ~1590 cm^−1^). It is well known that the D band is related to the sp^3^ carbon structure derived from the structural defects or partially disordered graphitic domains, while the G band is associated with sp^2^ carbon structures (graphitic carbons) resulting from the in-plane vibration of carbon atoms. Furthermore, the intensity ratio of the D band to the G band (designated as *I*_D_/*I*_G_) can be selected as a typical parameter, which has been widely used to evaluate the defect degree of graphene and its oxidized derivatives [42]. In comparison with the *I*_D_/*I*_G_ of GO (1.32), RGO-*g*-BO shows a larger *I*_D_/*I*_G_ value of 1.44, implying that some extra defects were generated on the rGO surface because of the chemical grafting reaction. This result is also in good agreement with the conclusion reported in previous studies [9].

The phase structures of GO and RGO-*g*-BO were also investigated by XRD measurement, as presented in Figure 1c. For GO, a typical (001) diffraction peak at 2*θ* = 11.8° can be detected, showing an interlayer space (*d*-spacing) of 0.73 nm calculated using the Bragg equation [43]. Compared to GO, the (001) diffraction peak in RGO-*g*-BO displays a significant shift from 11.8° to 10.3°, corresponding to an improved *d*-spacing of 0.84 nm, compared with that of GO (0.73 nm). This result can be easily explained by the fact that the introduction/grafting of BO onto GO surfaces enhances the interlayer distance of the graphene oxide lamellas in RGO-*g*-BO, further verifying the successful surface grafting of BO. Moreover, a new broad diffraction peak (002) was detected at 2*θ* = 22.1° in RGO-*g*-BO, which can be ascribed to the disordered self-restacking of partially reduced GO nanosheets during surface grafting and chemical reduction. Accordingly, based on the above discussion, the long-chain alkane grafting and partial chemical reduction of RGO-*g*-BO were successfully accomplished through both surface grafting reaction and in situ chemical reduction, paving the way toward enhanced lipophilicity and dispersion stability of RGO-*g*-BO additive in lubricants.

### 2.2. Microstructural Morphology and Particle Size Characterization

**Figure 2:**
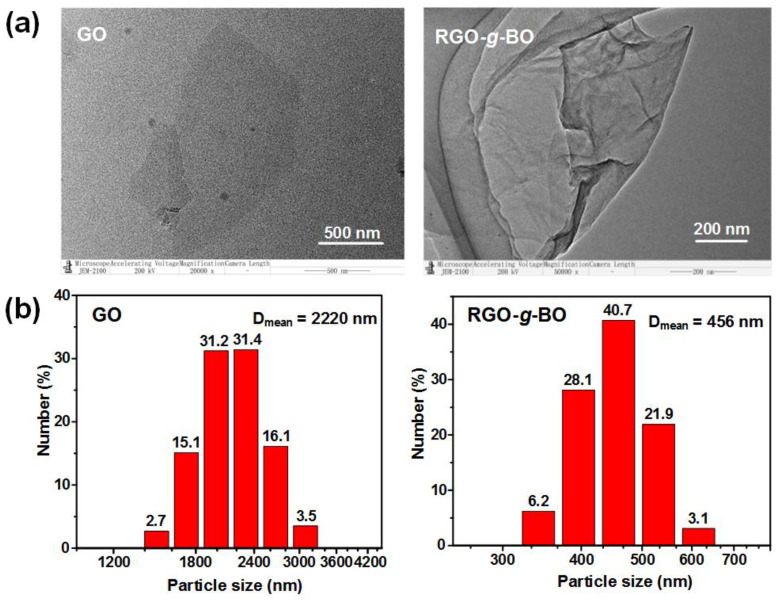
TEM images (**a**) and particle size distributions (**b**) of pristine GO and RGO-*g*-BO.

It is well established that the microstructural morphology and particle sizes of GO-based nanoadditives usually play an important role in their dispersion ability associated with the lubrication performance. In this work, the TEM and DLS measurements of GO and RGO-*g*-BO were carried out. As can be seen from Figure 2a, the TEM image shows that the larger GO nanosheets seem to be extremely transparent, suggesting a smaller thickness of GO nanosheets due to the efficient oxidation–exfoliation preparation process. In contrast, for RGO-*g*-BO, the leaf-shaped flakes show a more crumpled and stacked morphology, which can be ascribed to the enhanced Van der Waals force between the graphene-based sheets owing to the effective eliminating of the vast majority of the oxygen-containing groups driven by the chemical reduction process.

Additionally, the particle size distributions of GO and RGO-*g*-BO are presented in Figure 2b. It can be seen that the particle size of GO is mainly distributed in the range of 1400 to 3200 nm, whereas the particle size distribution of RGO-*g*-BO mainly ranges from 320 to 650 nm, and the average particle sizes of GO and RGO-*g*-BO are 2220 and 456 nm, respectively. Such small particle size of RGO-*g*-BO is believed to originate from the effective mechanical disruption by virtue of the high-speed dispersing and strong shear forces.

### 2.3. Thermal and Dispersion Stability

**Figure 3:**
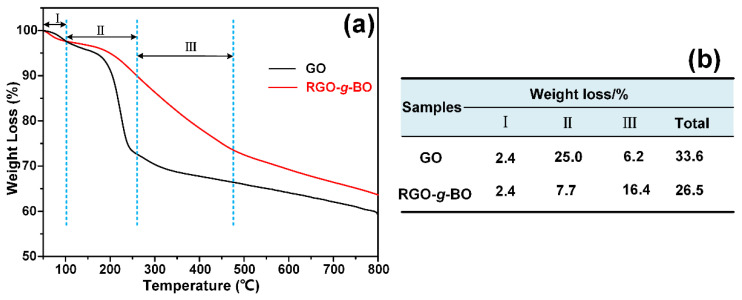
(**a**) Thermogravimetric analysis (TGA) results of GO and RGO-*g*-BO; (**b**) the summary of weight loss values of the tested samples within different decomposition temperature regions.

In order to assess the thermal stability of the as-obtained GO-based additives, a TGA test of the nascent GO and RGO-*g*-BO was carried out. As illustrated in Figure 3a,b, for GO and RGO-*g*-BO, 2.4% weight loss occurs in the first stage (I) ranging from 25 °C to 100 °C, which is believed to originate from the removal of the adsorbed free water in materials. In the second stage (II), ranging from 100 °C to 260 °C, 25.0% weight loss can be clearly seen from the decomposition curve of GO, which is mainly attributed to the decomposition of most of the oxygen groups in GO, including carboxyl, hydroxyl, and epoxy (-COOH, -OH and -C-O-C-). Compared to GO, a much lower weight loss of 7.7% is achieved for RGO-*g*-BO in the second stage (II), implying that the vast majority of oxygenated functional groups were eliminated due to the in situ chemical reduction procedure by virtue of sodium hydroxide. Moreover, a 6.2% weight loss is observed for pristine GO in the third stage (III), ranging from 260 °C to 475 °C, which might be attributed to the elimination of other more stable functional groups. However, a higher weight loss (16.4%) is observed for RGO-*g*-BO in the third stage (III), mainly derived from the decomposition of long-chain alkyl groups (i.e., C16). More importantly, the total weight loss of RGO-*g*-BO (26.5%) in the whole measurement range is less than that of GO (33.6%). Consequently, the above results convincingly verify that the successful surface grafting of long-chain alkane BO and the in situ chemical reduction process render RGO-*g*-BO with more remarkable thermal stability.

**Figure 4:**
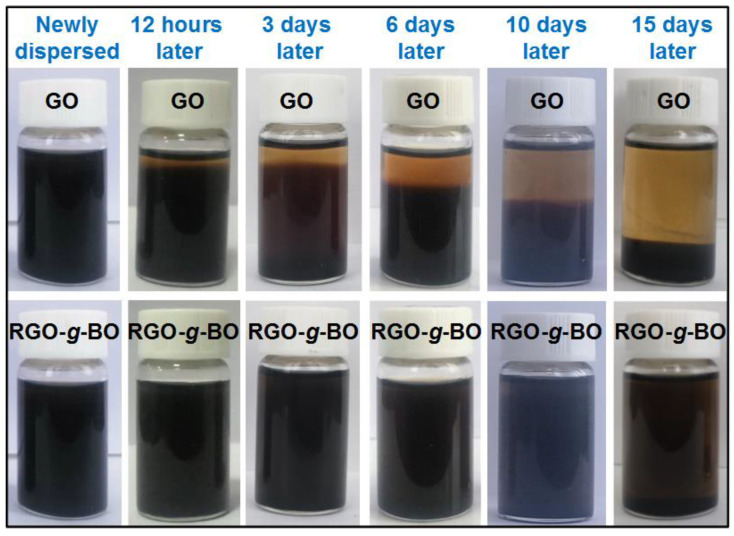
Dispersion stability evaluation of finished oil dispersed with pure GO and RGO-*g*-BO; the mass fraction of these two additives in lubricants is 0.005 wt%.

It is well known that the excellent dispersion capability and stability of additives in lubricating oils play a vital role in their high efficiency and durable lubrication application. Hence, the long-term dispersion stability of GO-based nanoadditives in the finished oil was inspected by the standing measurement. In this work, a kind of Castrol commercial oil was selected as the lubricant base oil. As shown in Figure 4, it is clearly seen that the GO and RGO-*g*-BO additives with a larger content (mass fraction, 0.005 wt%) are evenly dispersed in the commercial finished oil as a result of sonication treatment. For the finished oil with 0.005% GO, the GO additive tended to agglomerate and sediment from the lubricant matrix merely after 12 h. With the increase in time, the settlement phenomenon of the GO additive became more severe, which is subjected to the gravity and attraction force between graphene layers. After 15 days, the significant sedimentation layer can be clearly seen for the finished oil with GO. These results suggest that GO acting as an additive shows very poor dispersion stability in the finished oil system, which is mainly attributed to its ultralow lipophilicity because of its numerous oxygen-containing groups. Interestingly, the long-chain alkane functionalized RGO-*g*-BO nanoadditives display a dramatically improved dispersion capacity and remarkable dispersion stability. Specifically, the finished oil added with RGO-*g*-BO still maintained an ideal uniformity without any obvious precipitate behavior even after 15 days. This excellent dispersion stability of RGO-*g*-BO can be ascribed to the remarkable cooperative effects between the greatly enhanced lipophilicity of graphene additives as a result of the surface grafting and deep chemical reduction of long-chain alkane (BO) (as evidenced by the TGA results) and the desirable small-size effect (RGO-*g*-BO, *D*_mean_ = 456 nm).

### 2.4. Tribological Performance Evaluation

**Figure 5:**
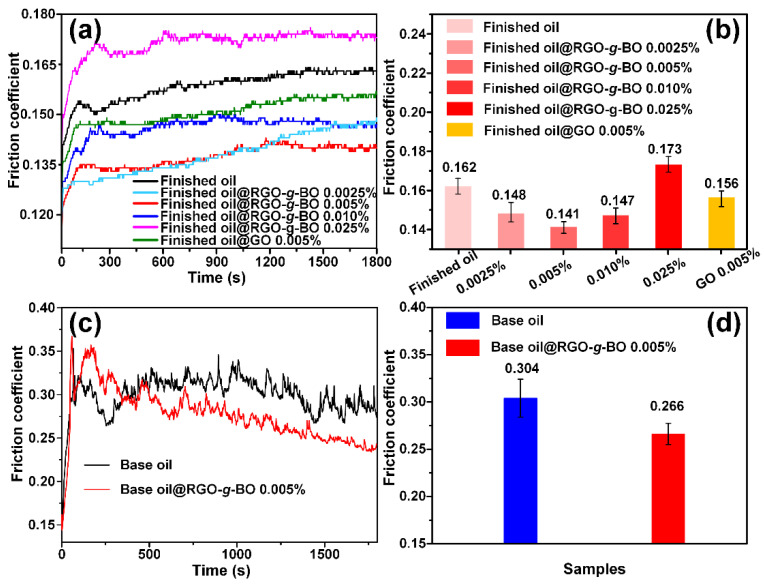
Friction coefficient variations as a function of time (**a**,**c**) and average friction coefficient **(b**,**d**) of pure finished oil, base oil, and their corresponding composite oil systems added with different weights of RGO-*g*-BO.

Encouraged by the remarkable dispersion stability of RGO-*g*-BO in the simulated finished oil, the tribological performances of the resultant graphene-based nanomaterials when used as lubricant additives were further examined, as shown in Figure 5. Figure 5a presents the changes in the friction coefficient values of finished oil filled with different weight fractions of RGO-*g*-BO with the measurement time. For nascent finished oil, a proper friction coefficient of 0.162 was achieved. In contrast, with the introduction of RGO-*g*-BO nanomaterial additives, the finished oil added with RGO-g-BO yielded significantly reduced friction coefficients compared to the pure finished oil. Meanwhile, it can be clearly seen that the friction coefficient/friction-reducing performance of finished oil filled with the RGO-*g*-BO nanolubricant is highly related to nanoadditive contents. Specifically, with the increase in RGO-*g*-BO mass fractions, the friction coefficient of a corresponding composite finished oil first decreased when the content of RGO-*g*-BO was lower than 0.005 wt%; however, it increased reversely after the concentration of RGO-*g*-BO exceeded 0.005 wt%. Apparently, the finished oil containing 0.005 wt% RGO-g-BO achieved the best friction-reducing performance, showing the lowest friction coefficient value of 0.141. Together with the excellent dispersion stability/durability of finished oil filled with 0.005 wt% RGO-*g*-BO, we can definitely conclude that the optimum additive content of RGO-*g*-BO was 0.005 wt% in the current work. It is worth noting that the friction coefficient of finished oil added with 0.025 wt% RGO-*g*-BO yielded a higher value (0.173) than that of the pure finished oil (0.162). Such seemingly contradictory results can be explained by the fact that excessive RGO-*g*-BO nanomaterials tend to agglomerate between friction pairs and break the continuity of the oil film, thus resulting in the occurrence of dry friction along with poor lubrication performance. Moreover, at the same additive content of 0.005%, the friction coefficient of finished oil added with pure GO was 0.156, much higher than that of the finished oil filled with RGO-*g*-BO nanolubricant (0.141).

In addition, to preclude the influence of other additives like antifriction agents and antiwear agents in finished oil, the pure base oil (i.e., hydraulic oil) without any additives, serving as a control sample, was also used to evaluate the corresponding tribological performance. As can be seen from Figure 5c,d, an extremely high friction coefficient of 0.304 was obtained for the base oil, which is around twice as much as that of pure finished oil (0.162). Interestingly, after the introduction of RGO-*g*-BO with an ultralow content (0.005 wt%), the base oil @ RGO-*g*-BO 0.005 wt% had a greatly reduced friction coefficient of 0.266, suggesting that RGO-*g*-BO can render the lube oil a prominent friction-reducing performance even with the absence of other functional nanoadditives. Therefore, based on the above discussion, RGO-g-BO nanomaterials derived from long-chain alkane lipophilic modifications and in situ chemical reduction onto GO have superior tribological properties.

**Figure 6:**
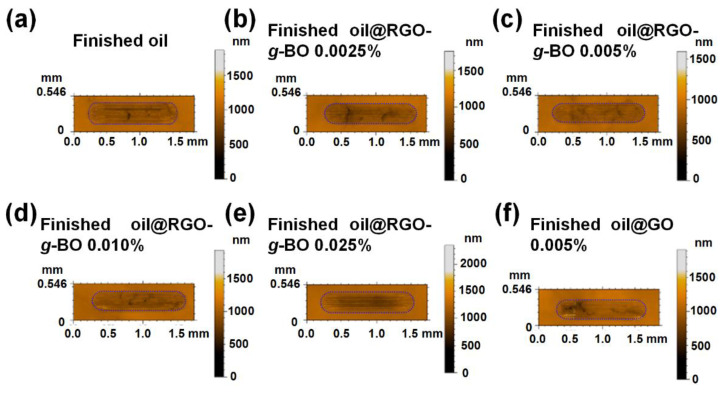
Surface morphologies of the rubbed scars on the steel disks after the friction tests lubricated with the pristine finished oil (**a**), finished oil filled with different concentrations of RGO-*g*-BO (**b**–**e**), and finished oil added with 0.005 wt% GO (**f**).

Apart from the friction-reducing effect, the wear-resistance properties of RGO-*g*-BO nanolubricants were also examined by measuring the corresponding wear parameters of wear scars. Surface morphologies, wear volumes, and surface roughness values of wear scars on the worn surfaces lubricated by nascent finished oil and finished oils filled with different contents of RGO-*g*-BO are presented in Figure 6 and Table 1. As seen from Figure 6a and Table 1 and Table 2, for the worn surfaces lubricated with pristine finished oil, many severe wear tracks such as deep grooves can be observed, implying a significant wear phenomenon. Moreover, relatively higher wear volume (52,151 μm^3^) and surface roughness (0.074 μm) were achieved for the rubbing surfaces with the presence of pristine finished oil. Apparently, as for the lubrication system of finished oil filled with pure GO, a distinct reduction in wear track areas was observed (Figure 6f) along with a decline in wear volumes (−28% increment, Table 1) and surface roughness values (−5% increment, Table 2) as compared to pure finished oil, indicating that a pure GO nanoadditive can improve the wear-resistance property of lubricating oil to some extent. By contrast, the finished oil incorporated with different RGO-g-BO additives was found to have a significantly enhanced antiwear performance, as evidenced by the decreased wear scare areas, wear volumes, and surface roughness values compared with those of pristine finished oil.

Notably, the enhancing effect of RGO-g-BO for finished oil in wear-resistance performance also exhibited a content dependency feature, and the corresponding characteristic parameters showed a similar change trend as that of the friction coefficients, as discussed above. More specifically, with the increase in the additive content of RGO-g-BO, the wearing parameters (including the wear track area, wear volume, and surface roughness of wear scars) first decreased (the content below 0.005 wt%) and then reversely increased. It can be clearly deduced that the finished oil containing 0.005 wt% RGO-*g*-BO had the best antiwear performances showing a relatively small and blurry wear area, the smallest values of the wear volume (24,391 μm^3^), and surface roughness (0.049 μm), which were decreased by 53% and 34% compared with the pristine finished oil, respectively. It should be pointed out that the finished oil filled with 0.025 wt% RGO-*g*-BO exhibited a degraded antiwear performance, with a significantly increased wear volume and surface roughness compared with pristine finished oil, which is mainly due to the excessive content of RGO-*g*-BO nanoadditives, as discussed above. Moreover, compared with GO, even with the addition of the same amount of 0.005 wt% to the finished oil, the RGO-*g*-BO system had a much more remarkable antiresistance performance, demonstrating the high effectiveness of graphene with better lipophilicity/dispersibility in improving the tribological properties. Accordingly, together with the excellent friction-reducing property, we can unquestionably conclude that the incorporation of RGO-*g*-BO in finished oil (even with an extremely low content of 0.005 wt%) can endow the as-prepared finished oil with a dramatically enhanced friction-reducing and antiwear performance, revealing its remarkable application potential in the lubricant additive field.

### 2.5. Exploration of Friction-Reducing and Antiwear Mechanism

In order to tentatively illuminate the corresponding friction-reducing and antiwear mechanism of graphene-based nanoadditives, the deposition behavior of lubricating oil components on the rubbing surfaces was first determined by systematical characterization using SEM, EDX, and Raman spectra on the corresponding wear scars, and the results are presented in Figure S1. Clearly, compared to the wear surface lubricated by pure finished oil, the counterpart derived from the lubrication of finished oil filled with RGO-*g*-BO (0.005 wt%) shows a greatly decreased wear state with very few wear scratches and shallow grooves, further demonstrating the excellent antiwear performance of RGO-*g*-BO nanolubricant. Moreover, from the EDX results presented in Figure S1(a2,b2), it can be inferred that the lubricant oil (finished oil components) exists on the rubbing surfaces since there are C, N, O, and Fe elements on both of the two worn surfaces. Nevertheless, this result fails to reveal the presence of the RGO-g-BO lubricant additives on the worn surfaces. Fortunately, the Raman spectra measurement of the wear scars illustrated in Figure S1(a3,b3) confirms the effective deposition of RGO-g-BO on the rubbed regions during the tribological testing process. Particularly, the characteristic peaks of graphene-based nanoadditives, including the D band (1320 cm^−1^) and the G band (1645 cm^−1^), can be clearly detected in the Raman spectrum of wear scar applied with finished oil containing RGO-*g*-BO, while there is no characteristic peak in the Raman spectrum for the wear scar treated by pure finished oil. Overall, such distinct results demonstrate that graphene-based additives (RGO-*g*-BO) indeed enter the rubbing surfaces during the friction process, which plays a crucial role in enhancing the tribological performance of lubricating oils.

**Figure 7:**
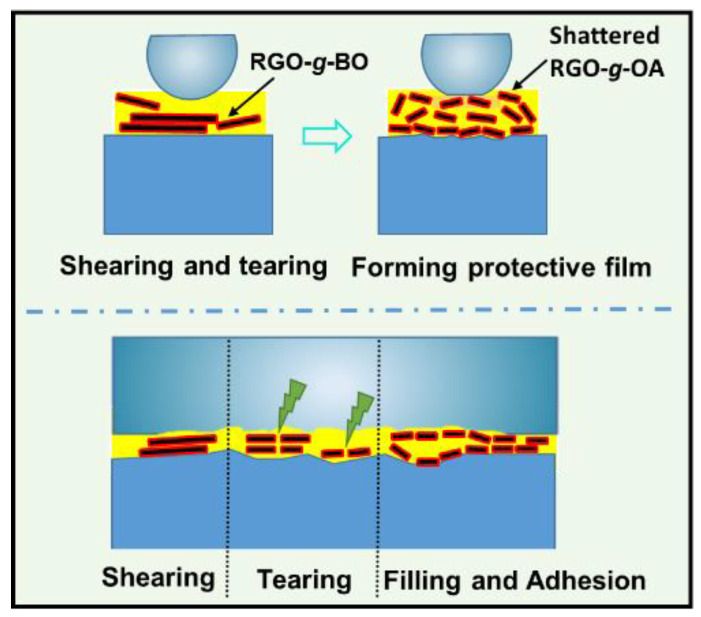
Schematic of friction reduction and antiwear mechanism of the finished oil filled with RGO-*g*-BO.

Based on the abovementioned results, the mechanism regarding the excellent friction-reducing and antiwear properties of RGO-g-BO nanomaterials can be elucidated as follows: Benefiting from the pony-size and ultrathin lamellar structural features, as evidenced in Figure 2, RGO-*g*-BO nanoadditives are capable of easily entering into the rubbing surfaces between the friction ball and steel disc, thus facilitating lube oils to form an intermittent and thin oil film in the initial stage of the friction process. Due to the significant shearing and tearing action during the violent friction process, these RGO-*g*-BO materials tend to become much smaller shattered nanosheets (as depicted in Figure 7); in this case, RGO-*g*-BO additives can fully fill the holes and grooves produced onto the bottom steel disc, hence enabling the lubricant oil to form a consecutive, thick and stable tribological protective film so as to restrain the direct contact of friction pairs. Notably, the inherent excellent mechanical strength of the graphene-based nanoadditives can afford a robust impact-resistant layer, thus effectively offsetting the external heavy wear behavior. Overall, thanks to the robust and successive tribological shielding film, the finished oil introduced with RGO-*g*-BO nanomaterials can thus render the surfaces remarkable friction-reducing and wear-resistance properties during the friction process.

## 3. Experimental Procedures

### 3.1. Materials

Natural graphite (purity > 99%, 300 mesh) was obtained from Qingdao Haida Co., Ltd. Concentrated sulfuric acid (H_2_SO_4_, 98%), potassium permanganate (KMnO_4_), sodium nitrate (NaNO_3_), hydrogen peroxide (H_2_O_2_, 30%), hydrochloric acid (HCl, 37%), ethanol, and sodium hydroxide (NaOH) were purchased from Sinopharm Chemical Reagent Co., Ltd. (Beijing, China) Bromooctadecane (BO) was supplied by Tianjin Bodi Co., Ltd. (Tianjin, China). Tetrabutylammonium bromide (TBAB) was obtained from Tianjin Fuyu Fine Chemical Co., Ltd. (Tianjin, China). Ultrapure water (UP, 18.25 MΩ) was produced with the ULTRAPURE (UPHeI-20T). The hydraulic oil (kinematic viscosity, 13.2 mm^2^/s at 40 °C) was provided by Kunlun Lubricant (Handan, China) and used as the base oil. The finished oil utilized in this work was synthesized in the laboratory.

### 3.2. Fabrication of Chemically Functionalized Reduced Graphene Oxide (RGO-g-BO)

Initially, graphene oxide (GO) was synthesized according to a typical modified Hummers method [9]. As presented in Scheme 1, the resultant GO was then fragmented with ultrasonication for 15 min, followed by homogenization dispersion treatment using a homogenizer (XHF-DY) at 4500 r/min for 15 min (repeated three times) to obtain pony-size graphene oxide (PGO) nanosheets. Afterward, the chemically functionalized reduced graphene oxide was prepared by surface grafting long-chain bromooctadecane (BO) using a simple and facile one-pot method. More specifically, the above PGO (50 mg) and UP water (100 mL) were added to a dried three-necked round-bottom flask (250 mL) and fully mixed with ultrasonic treatment for 30 min. After that, 10 mg of NaOH, 20 mL of the ethanol-solution-dissolved BO (2.5 mg/mL), and TBAB (50 mg) were added into the round flask with further mixing using ultrasonication (15 min). The surface grafting reaction was then conducted at 80 °C for 12 h in a nitrogen atmosphere. After the reaction, the as-obtained product was washed with a mass of ethanol by filtration three times and subsequently completely dried in a vacuum oven at 60 °C for 6 h to obtain the long-chain alkylated reduced graphene oxide (RGO-*g*-BO).

### 3.3. Preparation of Diverse Lubricant Oil Samples

The as-obtained RGO-*g*-BO (75 mg) nanosheets were first entirely dispersed in 30 mL of THF (2.5 mg/mL) with ultrasonication treatment (40 min). Subsequently, the above GO-based additive mixture was added to a certain volume of lubricating oil (including finished oil or base oil) and then sufficiently magnetically stirred (800 rpm) for 25 min. After homogenous mixing, THF was completely removed using a rotary evaporator at 60 °C (~1 h). The weight contents of the RGO-*g*-BO additive in the finished oils were 0 wt%, 0.0025%, 0.005 wt%, 0.010 wt%, and 0.025 wt%, respectively, while the corresponding weight content in the base oil was maintained at 0.005 wt%. Moreover, as a control sample, the finished oil filled with 0.005 wt% of GO was also prepared using a similar method.

### 3.4. Tribological Property Evaluation

A tribological performance test was conducted using an Optimol SRV oscillating reciprocating friction and wear tester. The corresponding experimental device has been reported in our previous work [9]. Specifically, the diameter of GCr15 bearing steel ball was 10 mm with a surface roughness of around 20 nm, while the surface roughness of GCr15 bearing steel discs (size: 24 mm × 8 mm) was ~40 nm. For each test, the friction pair was fully cleaned in petroleum ether by virtue of ultrasonication treatment (15 min) in advance to remove the adhered organic contaminants. Subsequently, 25 μL of finished oil/base oil dispersed with different concentrations of graphene-based additives was uniformly added onto the steel disc surface. Note that a 30 s running-in period before each formal test was used to prevent the friction pair from damaging. The load was initially set as 50 N and then slowly increased to the eventually pre-designed value (e.g., 100 N). During the measurement process, all the samples were tested using the same conditions (a stroke of 1 mm, a load of 100 N, a frequency of 25 Hz, and a temperature of 50 °C). The corresponding friction coefficient values could be automatically recorded with a recorder assembled onto the SRV tester. After the tests, the above friction pairs were washed with petroleum ether through ultrasonication (15 min) to eliminate the residual lubricating oils and some occasionally accumulated debris. Afterward, the wear volumes of wear scars on the tested discs were determined with a MicroXAM 3D (KLATencor, Milpitas, CA, USA) noncontact surface mapping profiler (equipped with analysis software of Apex II).

### 3.5. Characterization

Fourier transform infrared (FTIR) spectra were recorded with a Bruker VERTEX 70 spectrometer (4000–400 cm^−1^, Bruker company, Karlsruhe, Germany). Raman spectra measurement was carried out using a Renishaw Invia Raman Microscope with a 532 nm laser excitation wavelength. X-ray diffraction (XRD) patterns were measured with a Bruker D8 X-ray diffractometer using Cu Kα radiation (λ = 1.54 Å, 2*θ* ranging from 5 to 60°). The microscopic morphologies of GO and RGO-*g*-BO were observed using a transmission electron microscope (TEM, JEM-2100, JEOL, Tokyo, Japan). Particle size distributions of pristine GO and RGO-*g*-BO sheets were determined through dynamic light scattering (DLS) using a Malvern Zetasizer Nano ZS90. Thermalgravimentric analysis (TGA) was characterized using a TA Instruments Q50 thermogravimetric analyzer under a nitrogen atmosphere (in the range of 50–800 °C at a heating rate of 10 °C/min).

## 4. Conclusions

In summary, a novel reduced graphene oxide (RGO-*g*-BO) nanoparticle additive featuring pony-size and long-chain alkyl groups was prepared to provide a highly efficient lubrication application through a facile one-pot approach consisting of surface grafting and in situ chemical reduction. The as-obtained RGO-*g*-BO nanoadditives exhibited excellent dispersibility and stability in the lubricating oil, which is mainly due to the enhanced oleophilic property of RGO-*g*-BO by virtue of the surface grafting of long-chain BO components on the GO. Thermogravimetric analysis (TGA) verified that the successful surface grafting of long-chain alkane BO, along with the in situ chemical reduction procedure, endowed RGO-*g*-BO with significantly improved thermal stability. More importantly, compared with pure finished oil, the finished oil filled with RGO-*g*-BO nanomaterial additives exhibited a dramatically enhanced friction-reducing and antiwear performance. Particularly, under the optimum additive content of as low as 0.005 wt%, the corresponding friction coefficient and wear volume markedly reduced to 0.141 and 24,391 μm^3^, decreasing by 13.0% and 53%, respectively, in comparison to the nascent finished oil. This superior lubrication performance is believed to originate from the successive tribology shielding film formed between the rubbing surfaces derived from small-sized RGO-*g*-BO nanoparticles, which can effectively inhibit the direct asperity contact and abrasion between the fraction modules. Therefore, in view of fascinating advantages such as excellent dispersion stability and durability, remarkable thermal stability, and prominent friction-reducing and antiwear performance, as well as simple preparation, this novel graphene-based nanomaterial holds great promise for high-performance lubrication application.

## Figures and Tables

**Table 1:** Wear volumes of the worn surfaces on the steel discs.

Samples	Wear Volume (μm^3^)	Increment
Finished oil	52,151	0%
Finished oil@RGO-*g*-BO 0.0025%	29,949	−43%
Finished oil@RGO-*g*-BO 0.005%	24,391	−53%
Finished oil@RGO-*g*-BO 0.010%	36,326	−30%
Finished oil@RGO-*g*-BO 0.025%	58,509	+12%
Finished oil@GO 0.005%	37,720	−28%

**Table 2:** Surface roughness of the worn surfaces on the steel discs.

Samples	Surface Roughness (μm)	Increment
Finished oil	0.074	0%
Finished oil@RGO-*g*-BO 0.0025%	0.055	−26%
Finished oil@RGO-*g*-BO 0.005%	0.049	−34%
Finished oil@RGO-*g*-BO 0.010%	0.068	−8%
Finished oil@RGO-*g*-BO 0.025%	0.085	+15%
Finished oil@GO 0.005%	0.070	−5%

## Data Availability

The data presented in this study are available on request from the corresponding author. The data are not publicly available due to protect the privacy of data and protect intellectual property rights.

## Supplementary Materials

The following supporting information can be downloaded at: https://www.mdpi.com/article/10.3390/molecules29092004/s1, Figure S1: SEM images, energy dispersion X-ray analyses (EDX), and Raman spectra of the wear scars on the steel discs after the friction tests lubricated with the pure finished oil (a, a1–a3) and finished oil added with 0.005 wt% of RGO-g-BO (b, b1–b3).
