# Supplementary material for: One-Pot Synthesis of Alkyl Functionalized Reduced Graphene Oxide Nanocomposites as the Lubrication Additive Enabling Enhanced Tribological Performance
Source: Molecules. 2024 Apr 26;29(9):2004. doi: 10.3390/molecules29092004 (PMC11085161; doi:10.3390/molecules29092004)
Supplement: Supplementary file 1 [file molecules-29-02004-s001.zip › molecules-2982918-supplementary.pdf]

Supporting Information

One-Pot Synthesis of Alkyl Functionalized Reduced Graphene Oxide Nanocomposites as the Lubrication Additive Enabling Enhanced Tribological Performance

Guangfa Zhang 1,*, Chao Zhu 1, Yehai Yan 1, Jian Cui 1 and Jingxian Jiang 2,*

1 Key Laboratory of Rubber-Plastics, Ministry of Education, Shandong Provincial Key Laboratory of Rubber-Plastics, School of Polymer Science and Engineering, Qingdao University of Science and Technology, Qingdao 266042, China; zhu22chao@qust.edu.cn (C.Z.); yh2223yan@qust.edu.cn (Y.Y.); jian2223cui@qust.edu.cn (J.C.)

2 School of Chemical and Environmental Engineering, Shanghai Institute of Technology, Shanghai 201418, China

* Correspondence: gfzhang@qust.edu.cn (G.Z.); jxjiang@zju.edu.cn (J.J.)

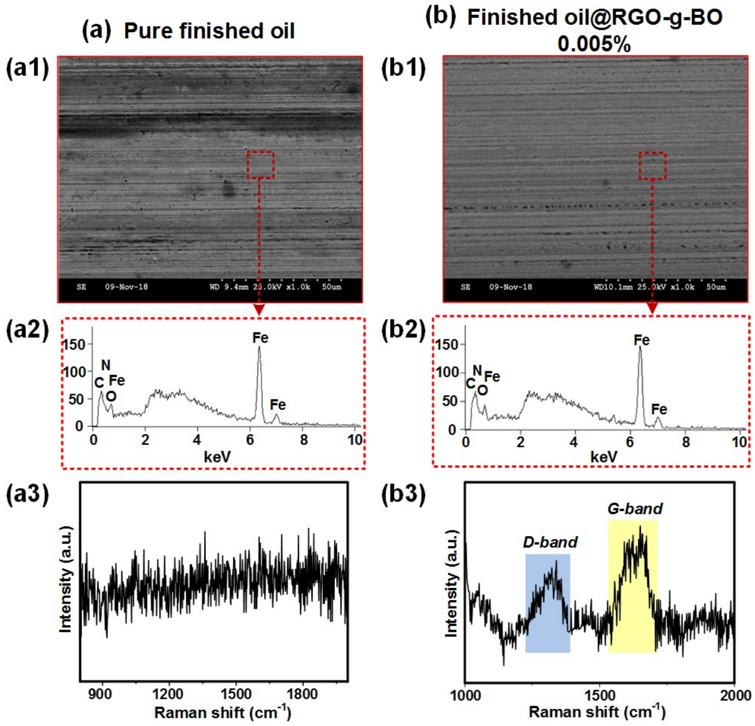

Figure S1. SEM images, energy dispersion X-ray analyses (EDX), and Raman spectra of the wear scars on the steel discs after the friction tests lubricated with the pure finished oil **(a, a1-a3)** and finished oil added with 0.005 wt% of RGO-*g*-BO **(b, b1-b3)**.
